# Virophage infection mode determines ecological and evolutionary changes in a host-virus-virophage system

**DOI:** 10.1093/ismejo/wrae237

**Published:** 2024-12-05

**Authors:** Ana del Arco, Lutz Becks

**Affiliations:** Aquatic Ecology and Evolution, Limnological Institute, University of Konstanz, Universitätsstraße 10, Konstanz/Egg 78464, Germany; Aquatic Ecology and Evolution, Limnological Institute, University of Konstanz, Universitätsstraße 10, Konstanz/Egg 78464, Germany

**Keywords:** eukaryotic cell, virophage, virus, chemostat, experimental evolution

## Abstract

Giant viruses can control their eukaryotic host populations, shaping the ecology and evolution of aquatic microbial communities. Understanding the impact of the viruses’ own parasites, the virophages, on the control of microbial communities remains a challenge. Most virophages have two modes of infection. They can exist as free particles coinfecting host cells together with the virus, where they replicate while inhibiting viral replication. Virophages can also integrate into the host genome, replicate through host cell division and remain dormant until the host is infected with a virus, leading to virophage reactivation and replication without inhibiting viral replication. Both infection modes (reactivation vs. coinfection) occur within host-virus-virophage communities, and their relative contributions are expected to be dynamic and context dependent. The consequences of this dynamic regime for ecological and evolutionary dynamics remain unexplored. Here, we test whether and how the relative contribution of virophage infection modes influences the ecological dynamics of an experimental host-virus-virophage system and the evolutionary responses of the virophage. We indirectly manipulated the level of virophage (Mavirus) integration into the host (*Cafeteria burkhardae*) in the presence of the giant Cafeteria roenbergensis virus. Communities with higher virophage integration were characterized by lower population densities and reduced fluctuations in host and virus populations, whereas virophage fluctuations were increased. The virophage evolved toward lower inhibition and higher replication, but the evolution of these traits was weaker with higher virophage integration. Our study shows that differences in the virophage infection modes contributes to the complex interplay between virophages, viruses and hosts.

## Introduction

Aquatic viruses play a central role for microbial community dynamics, biogeochemical cycling, and diversification [1–3]. Understanding how the role of viruses is influenced by their own parasites, the virophages, remains a challenge [4]. Many virophages can adopt a dual lifestyle in which they either integrate into the genome of the host of the virus, or they exist as free particles that can coinfect the host together with the virus [5]. Integrated virophages are transmitted vertically through the replication of the host. They can be reactivated upon viral infection of the host and parasitize the virus by using its virus factory for its own replication and the production of free virophage particles [6, 7]. During coinfection, the virophage also exploits the virus factory of the virus for its own replication, allowing for horizontal transmission. Although the importance of virophages for host and virus dynamics has been demonstrated [7–11], the ecological consequences of this viral exploitation by the virophage for the microbial community remain largely unknown.

The mode of virophage infection can be an important driver of ecological and evolutionary responses of host-virus-virophage dynamics. For example, coinfections in which virophages enter the host cell independently, as in the case of the Mavirus virophage [7], or in which they enter together with the virus, like the sputnik virophage [6], lead to different population dynamics [9]. When virophages can transmit vertically through host cell division or horizontally from one infected host cell to other host cells, as in the case of Mavirus virophages [5], the mode of infection affects virus replication: during reactivation of integrated virophages, virus replication is unaffected compared to infections in the absence of virophages, whereas during coinfections, virus replication is reduced or inhibited. These differences in virus replication have implications for population dynamics, e.g. by driving virus densities to very low levels [7].

In the absence of an infectious virus, the virophage’s mode of replication is fixed as vertical. In addition to vertical transmission with host replication, the virophage has two replication possibilities—reactivation of integrated virophage and coinfection – and they are expected to be present in a community if infectious virus is present. Their relative contributions may change depending on population densities, traits that determine the outcome of the interaction, and environmental conditions. Host, virus, and virophage densities change over time [7] affecting encounter rates between hosts and viruses and thus the likelihood for coinfections. In addition, traits underlying virus and virophage replication and host exploitation may evolve rapidly. For example, previous work with the Cafeteria-CroV-Mavirus system has shown that the high degree of exploitation and interdependence in this tripartite system leads to the rapid evolution of reduced replication of the virus (CroV) and increased virophage (Mavirus) replication, but reduced exploitation of the virus by the virophage [11]. These observations together suggest that the virophage infection mode is not constant over time and that the relative contribution of the two modes to virophage and virus reproduction may continuously change. The consequences of this dynamic regime for ecological and evolutionary dynamics are unexplored.

As a first step for developing an understanding of these consequences we test here the hypothesis that differences in the frequency of integrated virophage determine ecological (population dynamics) and evolutionary dynamics (virophage replication and viral inhibition; hereafter: exploitation by the virophage) through their effect on virus replication. We predict higher average virus and virophage densities in communities with higher levels of virophage integration because both viruses replicate after reactivation of integrated virophages. The long-term persistence of such a tripartite system should favor virophage traits that allow adaptations for sufficient exploitation of the virus without compromising the abundance of its viral host as a resource [12, 13]. This can be achieved if the virophage evolves higher levels of replication and lower levels of exploitation of the virus [11], as high levels of exploitation would result in low virus densities over time and thus few opportunities for the virophage to parasitize the virus. With more integrated virophages per host, we predict that selection for reduced exploitation will be weaker compared to hosts with fewer integrated virophages. This is because reactivation of integrated virophages leads to virus production and thus, on average, higher virus densities, whereas coinfection only leads to virophage production and thus lower virus densities. Because previous work has shown that increased reproduction and reduced virus exploitation by the virophage evolve together [11], we further predict that virophage reproduction will either not evolve or evolve with a smaller increase.

**Figure 1:**
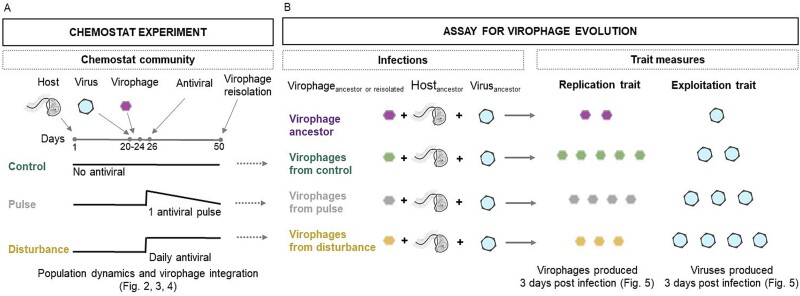
Experimental setup to test for and measures of evolutionary responses in the virophage when the level of integrated virophage into the host genome is indirectly manipulated. (A) Chemostats were assembled with the ancestral host (Day 1), ancestral virus and ancestral virophage (Day 20 and Day 24) and received no stressor (control), one application of the stressor (pulse) on Day 26, or daily application of the stressor from Day 26 (disturbance) until the end of the experiment (Day 50). From these chemostats we collected data on population dynamics and the level of integration of the virophage into the host genome. (B) To test for evolutionary responses of the virophage, we reisolated virophage from Day 50 of the experiment and compared the virophage traits replication and exploitation of the virus in short-term assays between ancestral and reisolated virophages from each chemostats during infections with ancestral host and ancestral virus. Depending on the stressor exposure, we predict different outcomes for trait evolution (see main text).

We tested the hypothesis in experiments with the host *Cafeteria burkhardae*, the giant virus Cafeteria roenbergensis virus (CroV) and the virophage Mavirus and manipulated the level of integration of the virophage into the host genome, by adding a chemical stressor to the experimental communities. Preliminary observations showed higher levels of virophage integration into the host genome resulting from an off-target effect of the antiviral oseltamivir (del Arco *personal observation*). We used the addition of the antiviral as an approach to manipulate the level of virophage integration rather than different host strains with naturally occurring variation in the number of integrated Mavirus. This allowed us to use the same genetic host background in all treatments. We tracked host, virus, and virophage population dynamics in replicate chemostat cultures (~150 host generations) under three different stressor levels that resulted in a gradient of integrated Mavirus per host cell: (i) control with no stressor added, (ii) pulse: one time application at Day 26 with the concentration of the antiviral which decreases over time due to the dilution rate of the chemostat system, (iii) disturbance: continuous application after Day 26. To look at the evolutionary consequences of differences in the frequency of integrated virophage, we compared virophage traits (replication and exploitation of virus by virophage) of ancestral (used to inoculate the chemostats) and evolved virophage (reisolated from the end of the experiment, Fig. 1).

## Materials and methods

The experimental system consisted of the host *Cafeteria burkhardae* (strain E4–10P, hereafter: host), the giant virus Cafeteria roenbergensis virus (CroV, hereafter: virus), and the virophage Mavirus (hereafter: virophage). All experiments were conducted using f/2 enriched artificial seawater medium [14] supplemented with 0.025% (w/v) yeast extract (hereafter: SW). The host strain E4–10P used to start the experiments carried endogenous virophages [15], but these are not expected to produce particles under the experimental conditions of this study [11].

### Manipulation of frequency of integrated virophage

We used the antiviral oseltamivir as an off-target antiviral to manipulate the frequency of the integrated virophage. The mode of action of oseltamivir is described as the inhibition of neuraminidase enzyme which is involved in Influenza A virus replication. Whereas the influenza virus and the viruses used here are fundamentally different (e.g. RNA vs. DNA viruses), we found that the presence of oseltamivir at a Predicted No Effect Concentration (PNEC) of 0.1 mg/L led to significantly higher integration of the virophage into the host genome while showing no short term effects on densities of host, virus, and virophage (see Supplementary Information, Fig. S1 and Fig. S2). Note, that it is out of the focus of this study to determine the molecular mechanism of how oseltamivir affects Mavirus and CroV. We used the PNEC as a benchmark for determining safe concentration levels of substances to protect ecosystems, aiming to mimic single (e.g. rainfall inflows) and continuous (regular discharges from wastewater treatment plants) exposure.

### Chemostat experiments

We assembled host-virus-virophage communities in chemostat systems with 400 ml of SW medium with a continuous flow through of 120 ml medium per day (= 0.3 dilution rate per day) [16]. Chemostats ran for 50 days in a culture room at 18 ± 0.5°C and served as an experimental setup that provided a controlled environment for manipulating stressor exposure within the microbial community (Fig. 1). Chemostats were inoculated with 7*10^3^ host cells/ml and isogenic virus and virophage were added after the host population dynamics had stabilized at Day 20. We inoculated the viruses again on Day 24 to ensure that the viruses established populations in all chemostats. Viruses and virophage were inoculated at a host:virus and host:virophage ratio of 0.1. The chemostats differed in the addition of the stressor to manipulate the integration of the virophage into the host genome. Four replicate chemostats received a one-time application of the stressor on Day 26 (hereafter: *pulse* treatment). Four replicate chemostats received a daily application of the stressor (hereafter: *disturbance* treatment). Four chemostat served as controls without the addition of the stressor. All chemostats received 2 ml of SW media, without or with antiviral (40 μl from a stock solution of 10^9^ ng/L), to meet the target stressor concentrations. Analytical grade stressor (Sigma Aldrich, oseltamivir phosphate ≥98% HPLC) was diluted in SW medium.

We sampled chemostats daily (sample = 5 ml, except 10 ml once a week) for quantification of densities. Host densities were quantified from life samples using a hemacytometer and light microscopy. Virus and virophage samples were frozen for later DNA extraction (DNeasy 96 Blood & Tissue Kit, Qiagen, Hilden, Germany) and quantification by digital ddPCR [11, 17]. All ddPCR results were analyzed using QUANTASOFT 1.7.4. The detailed methods and quality requirements for the data are described in the reference (18).

Stressor concentrations were measured in randomly selected replicates on two days: Day 26 right after the stressor pulse and the first addition to the disturbance treatment (four chemostats receiving the stressor pulse and one control) and Day 29 as part of the daily sampling (in two replicates of the pulse and three replicates of disturbance treatment). Samples were kept at 4°C and sent to SGS INSTITUT FRESENIUS GmbH (Radolfzell, Germany) to estimate concentration using gas Liquid Chromatography–Tandem Mass Spectrometry (LC–MS/MS). There was 0 mg/L in control chemostats, 0.11 ± 0.03 mg/L in the pulse and disturbance treatments after application (target concentration 0.1 mg/L). Three days after the first application, there was 0.05 ± 0.01 mg/L in the pulse and 0.16 ± 0.02 mg/L disturbance treatment.

### Integration of virophage into host genome

We measured virophage integration at Day 22 (2 days after the addition of viruses) and by the end of the chemostat experiment at Day 50. Virophage can be present in the community as free particles (horizontal transmission), or particle associated which can be either integrated virophage or virophage that is attached to organic particles. We estimated the particle associated fraction from the difference between total virophage abundance and virophage abundance after filtration through a 0.2 μm filter (Spartan cellulose filter; Whatman), which keeps the particles but let free virophage particles pass (Marvirus size ~70 nm [19]; for details see del Arco et al. 2022). As the fraction of virophage attached to organic particles was negligible in our experiments (see Supplementary Information, Fig. S3), we used the particle-associated virophage fraction to estimate the integrated virophage densities and from this the average number of integrated virophage per host (DNA copies per host).

To test whether host survival in the presence of virus was influenced by the level of virophage integration, we compared host survival between ancestral and reisolated clonal lines by the end of the experiment. For the latter, we isolated 10 clonal lines per chemostat from the last chemostat sampling day (Day 50) using serial dilutions. Specifically, samples were diluted to 300 cell/ml SW medium, and 1 μl of the diluted sample was pipetted into 200 μl medium in 96 well plates at 18 ± 0.5°C. Growth of cells was checked after 6 days, and positive samples transferred to in 20 ml of SW media in tissue culture flasks to establish cultures of clonal lines. We prepared isolated clones of ancestral hosts following the same protocol. We tested host survival by infecting the clonal cultures of the host (starting density 7*10^3^ host cells/ml) with virus at a host:virus ratio of 100 (n = 2). Infections were done in 96 well plates (total volume of 200 μl of SW media). After 5 days post infection, host samples were fixed with Lugol’s solution (4% final concentration). Host survival was estimated using a high content microscope (ImageXpress Micro 4; Molecular Devices, 20x magnification, transmitted light) and we applied a custom module with the MetaXpress software for cell counting. We used presence/absence of host cells as measure of survival.

### Virophage evolution

We tested whether and how virophage evolved during the chemostat experiment by comparing traits (virophage replication and exploitation) of ancestral and reisolated virophages from the end of the experiment (Fig. 1). By the end of the experiment, around 150 host generations had coexisted with both the virus and the virophage, making it possible to detect the evolutionary changes that occurred during this period. For the latter, we reisolated virophages from the chemostats at Day 50 by filtration through 0.2 μm filters. We then infected ancestral host in 200 μl of SW media in well plates with ancestral virus, and ancestral or reisolated virophage at a host:virus and host:virophage ratio of 0.5 and 0 (as control). All combinations were replicated four times and started with a host density of 10^3^ cells/ml. After 3 days post infection we quantified virus and virophage densities (as DNA copies/ml) using ddPCR (see above). We followed standard procedures in experimental evolution [11, 20] and performed assays under standardized conditions (i.e. independent of and several rounds of replication after reisolation) and were thus able to identify heritable phenotypic changes in the virophage populations.

### Reactivation of integrated virophage

In addition, we reisolated a total of 88 host clonal lines from the end of the experiment: 23 came from the control, 28 from the pulse treatment, and 37 from the disturbance treatment. We used serial dilutions from the treated samples for reisolation of host clonal lines. Specifically, samples were diluted to 300 cell/ml, and 1 μl of the diluted sample was pipetted into 200 μl medium in 96 well plates at 18 ± 0.5°C. Growth of cells was checked after 6 days, and positive samples transferred to 20 ml of SW media in tissue culture flasks to establish cultures of clonal lineages. As some cultures of the clonal lines contained free virophage, we only continued with those cultures, where we did not detect free virophage. We assessed virophage abundance following viral infection in ancestral and reisolated hosts. In replicated experiments (n = 3), we either added ancestral virus (virus:host ratio of 20) or left hosts without virus. At 5 days post infection, we quantified virophage DNA copies using ddPCR (see above), and differences in virophage abundance were estimated as reactivated virophage by comparing DNA copies from the virus-free controls and the treatment where we added virus.

### Data analysis

All data analyses were performed in Rstudio [21] and R [22] using the packages geepack [23] and multcomp [24]. Differences between models were considered relevant when *P* < 0.05. We tested for differences in virophage integration by using generalized linear models (GLMs) (family = poisson) with treatment (control, pulse, and disturbance) as explanatory variable. To test for differences in host survival, we compared the percentage of clones growing in each treatment in the presence of ancestral virus using generalized linear models (family = poisson) with treatment (control, pulse, and disturbance) as explanatory variable. We evaluated differences in virophage reactivation between the ancestor and selected host clones with integrated virophages. We used generalized linear mixed model (family = poisson) to compare between host lines (ancestral, control, pulse, and disturbance) and their interaction as explanatory variable. Amplitude and density of host, virus and virophage over the experiment were compared using GLMs (family = poisson) with treatment (control, pulse, and disturbance) as explanatory variable. Amplitude was calculated as the density differences between the minimum and maximum values observed during a cycle calculated over a 3-day interval. For the density comparison, we used the harmonic mean of it (i.e. type of average that gives more weight to lower values, aiming to averaging reciprocals as an alternative to the arithmetic mean which is less responsive to such values). To test for differences in the ratios of virus:host, virophage:host, and virus:virophage densities we used GLMs (family = poisson) with treatment (control, pulse, and disturbance) and time and their interaction as explanatory variable. We used wavelet analyses using the WaveletComp package [25] to estimate cycles length in population ratios of (a) virus-host, (b) virophage-host, and (c) virophage-virus for each community. For this, data were smoothed following standard analysis procedures. Cycle lengths were compared between treatments (control, pulse, or disturbance) using linear models. For all testes, we used Tukey’s posthoc tests corrected for multiple testing. Finally, to test for differences in virophage trait evolution (virophage replication and exploitation of the virus), we used GLMs (family = poisson) with virophage line (ancestor, coming from control, pulse, or disturbance treatment) as explanatory factor.

## Results

**Figure 2:**
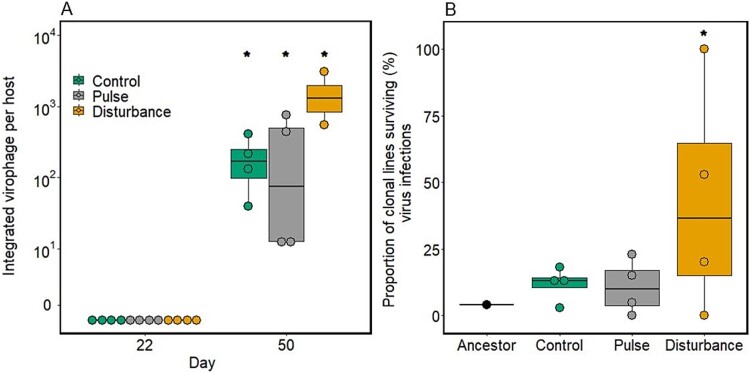
Virophage integration and host survival. (A) Virophage integration as copies of virophage DNA per host genome after Mavirus and CroV addition to the chemostat communities at Day 22, and at the end of the experiment at Day 50. (B) Survival of clonal host populations reisolated from Day 50 of the experiment when growing in the presence of the ancestral virus. Asterisks indicate statistical difference between treatments as determined by Tukey’s posthoc tests (*P* < 0.05).

We tested for the relative role of the two modes of virophage infection and manipulated the level of integrated virophage by adding a stressor. We tracked the population dynamics of host, virus, and virophage over 50 days (30 days after virus and virophage inoculation to host populations) in populations that differed in stressor manipulation, therefore in levels of integrated virophages, and compared the traits between the ancestral and reisolated virophage. To test whether the manipulation was successful, we estimated the number of integrated virophages per host on Day 22 after virophage and virus inoculation and before stressor manipulation, as well as on Day 50 at the end of the chemostat experiment. We found no integrated virophages on Day 22. At Day 50, virophage was integrated into the host genome in all communities (Fig. 2A, GLM, treatment: df = 2, *χ*^2^ = − 3379, *P* < 2.2*10^−16^) and the integration was significantly higher in communities with the higher stressor manipulation (Fig. 2A, post-hoc test for all pairwise comparisons: control vs. pulse vs. disturbance *P* < 2.2*10^−16^).

We tested whether host survival following virus infection was affected by virophage integration using ancestral host (without integrated virophages) and reisolated host clonal lines from Day 50 (see Methods for details, Fig. 1). The ancestral host clonal lines did not survive viral infections, whereas reisolated lineages coming from chemostat treatment did (Fig. 2B, GLM, host line: df = 3, *χ*^2^ = 136.41, *P* < 2.2*10^−16^). Specifically, reisolated host clonal lines from communities with continuous antiviral exposure and higher virophage integration (disturbance) showed higher host survival than those from the pulse and control treatments (Fig. 2B, post-hoc test: disturbance differs from all others, *P* < 2.2*10^−16^). We assessed virophage reactivation using host clonal lines with integrated virophage (four control, fifteen pulse, and five disturbance) and ancestral lines but we found no evidence of free virophage. We did not detect virophage reactivation in the ancestral host lines (Supplementary Information, Fig. S4, Generalized Linear Mixed model (GLMER) with chemostat as random factor representing the origin on the host lines, treatment: df = 3, *χ*^2^ = 38 707 731, *P* < 2.2*10^−16^). Virophage reactivation occurred in reisolated host lines. Virophage production thought reactivation of integrated virophages in host genome was lower as the level of stressor increased from control to the disturbance treatment (Fig. S4, post-hoc test: all comparisons *P* < 2.2*10^−16^).

### Population dynamics

**Figure 3:**
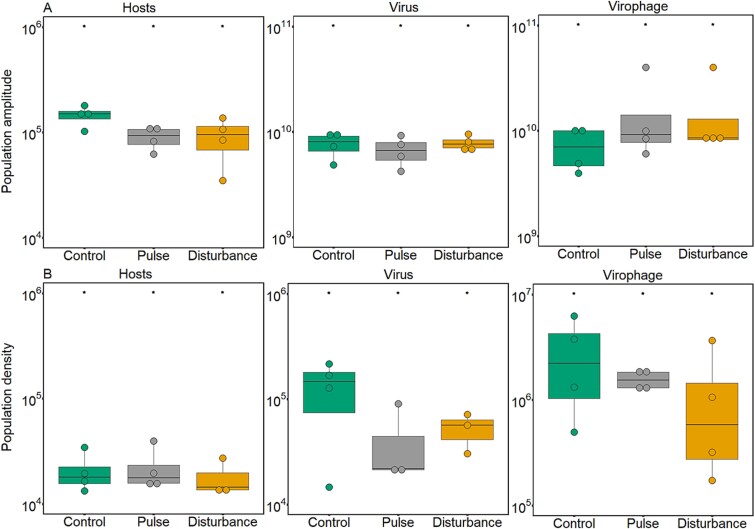
Host, virus, and virophage amplitude and density. (A) amplitudes of population cycles (host cell/ml, virus, or virophage DNA copies/ml for Days 26–50 of the chemostat experiment, i.e. after the beginning of the stressor treatments). (B) Harmonic mean densities (host cell/ml, virus, or virophage DNA copies/ml over Days 26–50 of the chemostat experiment). Asterisks indicate statistical differences between treatments as determined by Tukey’s posthoc tests (*P* < 0.05).

Population densities cycled after virus and virophage introduction independent of the presence of the stressor (Figs. 3, S5). In the host population, cycles differed in their amplitude (Fig. 3A, GLM, treatment: host, df = 2, *χ*^2^ = 69 881, *P* < 2.2*10^−16^). Amplitudes were significantly lower for the host populations in the presence of the stressor (Fig. 3A, post-hoc test: stressor manipulation *P* < 0.05). Amplitudes of the virus population were also lower in the presence of the stressor (Fig. 3A, GLM, treatment: virus, df = 2, *χ*^2^ = 411 946 915, *P* < 2.2*10^−16^) and significantly different between the treatments (post-hoc test: stressor manipulation *P* < 2.2*10^−16^). For virophages, we found significantly higher amplitudes than in host and virus populations (Fig. 3A, GLM, treatment: virophage, df = 2, *χ*^2^ = 1.8*10^10^, *P* < 2.2*10^−16^), being the higher in the disturbance treatment (post-hoc test: stressor manipulation *P* < 2.2*10^−16^). These differences in population dynamics, resulted also in differences in the harmonic means of the host, virus, and virophage populations. Specifically, in densities of host being the lower of the three populations (Fig. 3B, GLM, treatment: host, df = 2, *χ*^2^ = 6380, *P* < 2.2*10^−16^), followed by virus (Fig. 3B. GLM, treatment: virus, df = 2, *χ*^2^ = 321 638, *P* < 2.2*10^−16^), and virophage with the higher densities (Fig. 3B. GLM, treatment: virophage, df = 2, *χ*^2^ = 3 057 765, *P* < 2.2*10^−16^). In addition, population densities differed between treatments in the three populations with lower densities in the stressor treatments compared with the control (post-hoc test: stressor manipulation *P* < 2.2*10^−16^).

**Figure 4:**
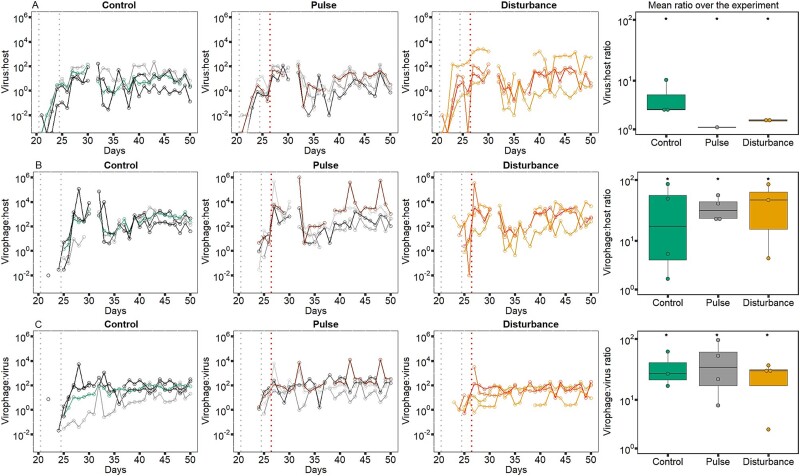
Population dynamics. Ratios of (A) virus and host, (B) virophage and host, and (C) virophage and virus in the control treatment (first column), in the pulse treatment receiving a one-time stressor addition on Day 26 (second column; red dotted line), and disturbance treatment where the stressor was added continuously after Day 26 (third column). The four replicates per treatment and ratio are presented by different shades (green: Control; grey: Pulse, orange: Disturbance). The grey lines represent virus and virophage addition. The last column of graphs represents the harmonic means of the ratio. Asterisks indicate statistical differences between treatments as determined by Tukey’s posthoc tests (*P* < 0.05).

Population ratios also differed between the communities, and they cycled over time with a significant period of 6 ± 1 days (Fig. 4) with no differences in cycle length between the treatments (LM, treatment: virus:host *χ*^2^ = 0.742, df = 2, *P* = 0.599; virophage:host *χ*^2^ = 1, df = 2, *P* = 0.405; virophage:virus, *χ*^2^ = 0.001, df = 2, *P* = 0.999). The virus:host ratios changed over time depending on the treatment (Fig. 4A, GLM, treatment × time: df = 2, *χ*^2^ = 15.943, *P* < 0.003, treatment: df = 4, *χ*^2^ = 21 039, *P* < 2.2*10^−16^, time: df = 3, *χ*^2^ = 90.424, *P* < 2.2*10^−16^) with lower average ratios in the presence of the stressor (post-hoc test: all comparison *P* = 0.0001). The virophage:host ratio differed also over time and depending on the treatment (Fig. 4B, GLM, treatment × time: df = 2, *χ*^2^ = 269 603, *P* < 2.2*10^−16^, treatment: df = 4, *χ*^2^ = 3 935 843, *P* < 2.2*10^−16^, time: df = 3, *χ*^2^ = 656 096, *P* < 2.2*10^−16^) with higher average ratios in the presence of the stressor (post-hoc test: all comparison *P* < 2.2*10^−16^). This also led to significant differences for the average virophage:virus ratios over time and between treatments (Fig. 4C, GLM, treatment × time: df = 2, *χ*^2^ = 5758.8, *P* < 2.2*10^−16^, treatment: df = 4, *χ*^2^ = 50 442, *P* < 2.2*10^−16^, time: df = 3, *χ*^2^ = 6773.4, *P* < 2.2*10^−16^) with significant higher ratios in the pulse treatment (post-hoc test: all comparison: *P* < 2.2*10^−16^).

### Virophage evolution

**Figure 5:**
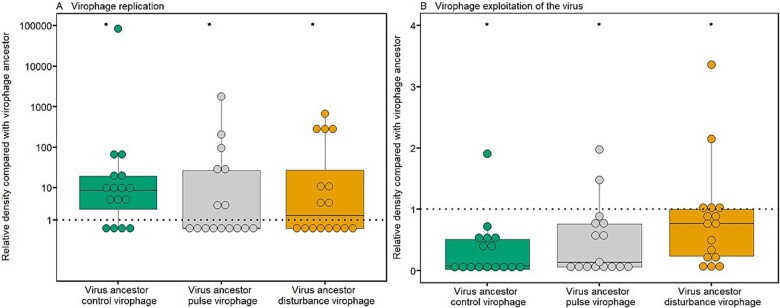
Virophage trait evolution. (A) Virophage replication. Virophage densities of reisolated virophage 3 days post infection (infections with ancestral host and virus). A value above 1 means that the reisolated virophages have increased replication compared to the ancestor. (B) Virophage exploitation estimated as its effect on virus replication. Virus densities 3 days post infection in the presence of reisolated virophage (infections with ancestral host and virus). A value below 1 means that the reisolated virophages have decreased virus exploitation compared to the ancestor. Each point represents an isolated virophage population coming from one of the four replicates per chemostat (trait assays with four replicates; total 16 virophages per treatment). Asterisks above bars indicate statistical differences between treatments as determined by Tukey’s posthoc tests (*P* < 0.05).

We tested whether and how virophage reproduction and exploitation evolved in the experiment by comparing these traits between virophages reisolated from Day 50 of the experiment and the virophage used to start the experiment. Reproduction of reisolated virophages from all chemostats treatments was higher compared to ancestral virophages (Fig. 5A, GLM: virophage line: *χ*^2^ = −46 743 054, df = 3, *P* < 2.2*10^−16^) and differed among the treatments, with decreasing reproduction with increasing stress level (post-hoc test: all comparison: *P* < 2.2*10^−16^). Therefore, in the presence of the stressor, the increase in replication relative to the ancestor is modulated by the level of integrated virophage derived from the manipulation with the antiviral (Figs. 1, 5). There is a decreasing gradient in virophage replication across treatments, with the highest replication levels in the control, followed by the pulse and then the disturbance treatments. Specifically, the increase in replication is lower in virophage populations with higher levels of integrated virophages. In contrast, the opposite trend is observed for virus exploitation, that is virophage exploitation of the virus was lower for reisolated virophages compared to ancestral virophages (Fig. 5B, GLM, virophage line: *χ*^2^ = −10 902 981, df = 3, *P* < 2.2*10^−16^) and lower despite of higher virophage replication of virophages in selected virophages (post-hoc test: all comparison: *P* < 2.2*10^−16^, Fig. 5A). In this case, virus exploitation levels were lowest in the disturbance treatment, followed by the pulse treatment, and then the control treatment.

## Discussion

The dual nature of the virophage lifestyle is unique among eukaryotic DNA viruses and provides the virophage with complex ways to interact with the virus and the host cell with distinct effects on the virophage and virus reproduction. The relative roles of different infection modes can change with conditions, and their shifts may play a central role in the ecological and evolutionary dynamics of microbial communities because the mode of virophage replication affects virus replication. Here, we tested the ecological and evolutionary consequences of different virophage infection modes by indirectly manipulating the frequency of integrated virophages per host cell. We observed that virophages evolved higher levels of replication but lower levels of exploitation over time in all treatments, as also previously observed in this system in the absence of a stressor [11]. Compared to the ancestor the increase in replication and decrease in exploitation of the virus was less pronounced at higher levels of virophage integration. This is consistent with the prediction for the effect of higher levels of integrated virophage on virophage evolution. Low levels of integrated virophage, in the absence of the stressor, impose stronger selection on the virophage for increased replication and lower exploitation. Increased virophage replication leads to coinfection and subsequent viral inhibition, which can ultimately lead to viral extinction and a decline in virophage populations. Under this scenario evolution favors reduced virophage exploitation of the virus and higher virophage replication. Differences in virophage traits will influence the impact of the infections mode (reactivation vs. coinfection) on host-virus-virophage interactions and population dynamics [9]. For instance, a shift in virophage traits toward higher replication increases the likelihood of coinfections, which in turn inhibit viral replication. This may enhance the protective role of the virophage for the host population and could potentially drive the virus to extinction, ultimately changing the community structure. The pattern of increased replication and decreased exploitation shows a correlation between exploitation of the virus and virophage replication, where virophage with higher replication have lower virus exploitation. Trait correlations are often observed in microbial systems including viruses with strong effects on the evolutionary and ecological dynamics [26, 27]. For example, they can influence the rate of evolution of resistance mechanisms against multiple consumers [28], the direction of evolutionary change [29], and they can determine population dynamics directly and indirectly through eco-evolutionary dynamics [30]. Identifying trade-offs in virophages provides a new perspective on virus-virophage interactions, as understanding the ecological impact and evolutionary potential of viruses and virophages relies on knowledge of their life history traits.

Host, virus, and virophage populations showed significant and similar cycles over time across communities. Most studies modelling host-virus-virophage interactions find cyclic dynamics in their simulations [8, 9, 31], which stabilize, e.g. as a function of virophage inhibition of the virus [8, 31], and coinfection mode (together vs. sequential, 9). Reduced exploitation in consumer-resource systems can stabilize their dynamics, leading to smaller amplitudes or a shift towards steady-state dynamics [32]. A recent model analysis showed that increasing the inhibition of virophages on virus replication can have a stabilizing effect on the oscillatory dynamics [31]. Here we did not find a pattern suggesting stabilization even though we found differences in virophage exploitation of the virus. Instead, populations from all three treatments cycled. This observation could be explained by at least three non-exclusive mechanisms, that needs further studies. First, the trait differences we observe at Day 50 of the experiment might only reflect very recent evolutionary changes, which may represent a transient evolutionary state over the number of generations covered in our study and differences in population dynamics might only be observed over longer experimental timescales in tri-partite systems compared to predator–prey systems [33, 34]. Second, the relative roles of virophage reproduction modes—vertical transmission, coinfection, or reactivation of integrated virophage—changed over time as a function of stressor treatments and population dynamics, and the consequences for population dynamics have not been fully explored theoretically [5, 8, 31]. Finally, stabilization with reduced exploitation may be less common in systems with such complex interactions as the one of the virophage-virus-host, and the parameter space explored in models so far does not cover some of the key biological parameter ranges [31]. Models capable of fitting experimental population trajectories could help to identify the underlying mechanisms governing the structure and function of host-virus-virophage communities. For example, they could help predict which population dynamics are indicative of the community transitioning from a region of coexistence to extinction as a function of infection mode [9], or disentangle the effects of trait changes on interaction strength within populations while maintaining similar population densities [35]. The cyclic dynamics could also explain the discrepancy between observed and predicted virus and virophage densities in communities with higher levels of virophage integration. We predicted that virophage and virus densities would be higher because virophages and viruses replicate after virophage reactivation. We found that virophage and virus densities were lower in communities with higher levels of integration. Whether this is a consequence of the specific biology of this system (different infection modes with different replication rates) or a general pattern found in host-virus-virophage systems needs to be investigated.

The ddPCR protocol used in this study targets the virophage Mavirus, but its efficiency to detect other endogenous Mavirus-like elements (EMALEs) carried by *C. burkhardae* strain E4–10P (Hackl et al. 2021) has not been assessed. It is thus possible that other EMALEs have been reactivated affecting the ecological and/or evolutionary dynamics. Koslová et al. (2024) characterized the reactivation of EMALES in a collection of globally distributed Cafeteria populations and they found that reactivation was stochastic, inefficient and EMALE-virus specific (e.g. only one out of eight EMALEs reactivate upon CroV infection). For the host strain E4–10, which we used here, EMALE04 was reactivated by CroV infections but low copies were produced, and host died within several days after infection. However, EMALE04 can inhibit the virus in subsequent rounds of infection reaching similar inhibition levels of the virus population as Mavirus [36]. Whether and how EMALE04 reactivated and competed with Mavirus in our experiment is not known. Because EMALE04 reactivation has been shown to be stochastic, we would expect that EMALE04 reactivation and accumulation in the communities would lead to large variation across replicates within treatments, which we do not observe. Competition among virophages is unexplored, which is particularly compelling due to its potential implications for understanding the eco-evolutionary dynamics of host-viral-virophage populations.

Experimental and field data suggest that virophages are key players shaping the dynamics and evolutionary trajectories of host and virus populations [6, 7, 10, 18]. For instance, in natural communities, virophages that infect algae can influence natural population dynamics exacerbating blooms of Antarctic phytoplankton during summer periods by suppressing parasitic viruses [10]. There is, however, little known about how the environment influences the level of virophage integration and reactivation. We found that higher level of integration showed a positive correlation with higher host survival, negative correlation with virophage reactivation and with viral inhibition. As host-virus-virophage communities are characterized by a high degree of population interdependence, shifts in the relative roles of replication modes must be considered as a potential key mechanism influencing community ecological and evolutionary responses.

## Supplementary Material

SI_Fig_1_wrae237

SI_Fig2_wrae237

SI_Fig_3_wrae237

SI_Fig_4_wrae237

SI_Fig_5_wrae237

ISMEJ-D-24-01274_R4_SI_wrae237

4_DataBaseRepository_wrae237

## Data sharing

Date are available in the public data repository Zenodo (https://zenodo.org/records/13862446).
