# Supplementary figures and images for: Virophage infection mode determines ecological and evolutionary changes in a host-virus-virophage system
Source: ISME J. 2024 Dec 5;18(1):wrae237. doi: 10.1093/ismejo/wrae237 (PMC11653005; doi:10.1093/ismejo/wrae237)

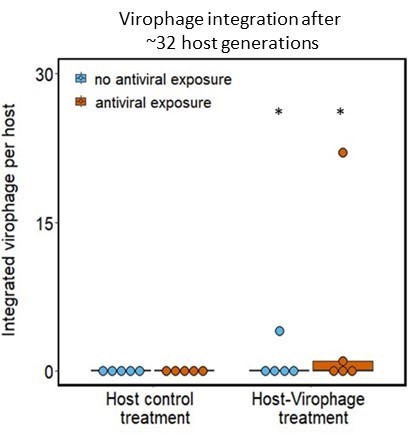

Supplement: SI_Fig_1_wrae237 [file si_fig_1_wrae237.jpeg]

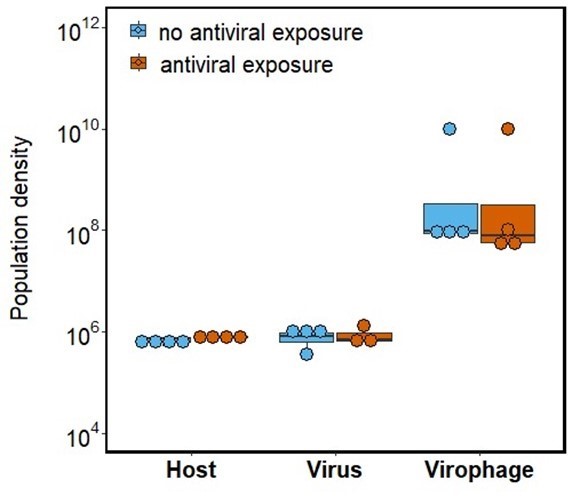

Supplement: SI_Fig2_wrae237 [file si_fig2_wrae237.jpeg]

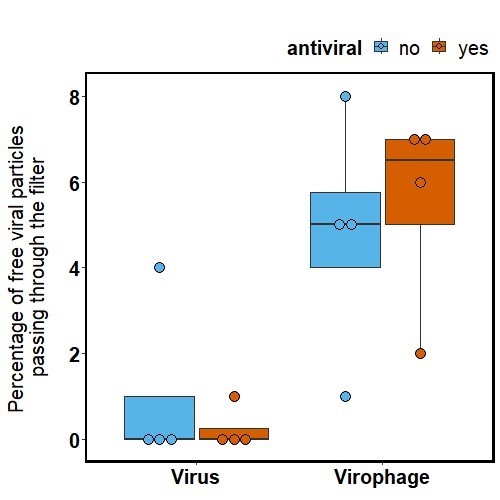

Supplement: SI_Fig_3_wrae237 [file si_fig_3_wrae237.jpeg]

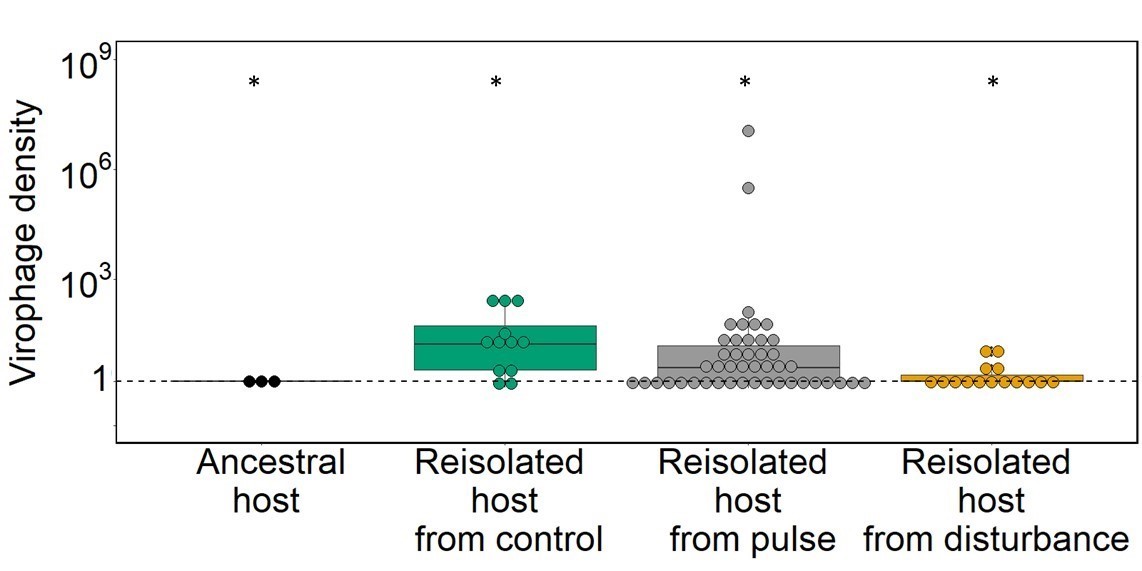

Supplement: SI_Fig_4_wrae237 [file si_fig_4_wrae237.jpeg]

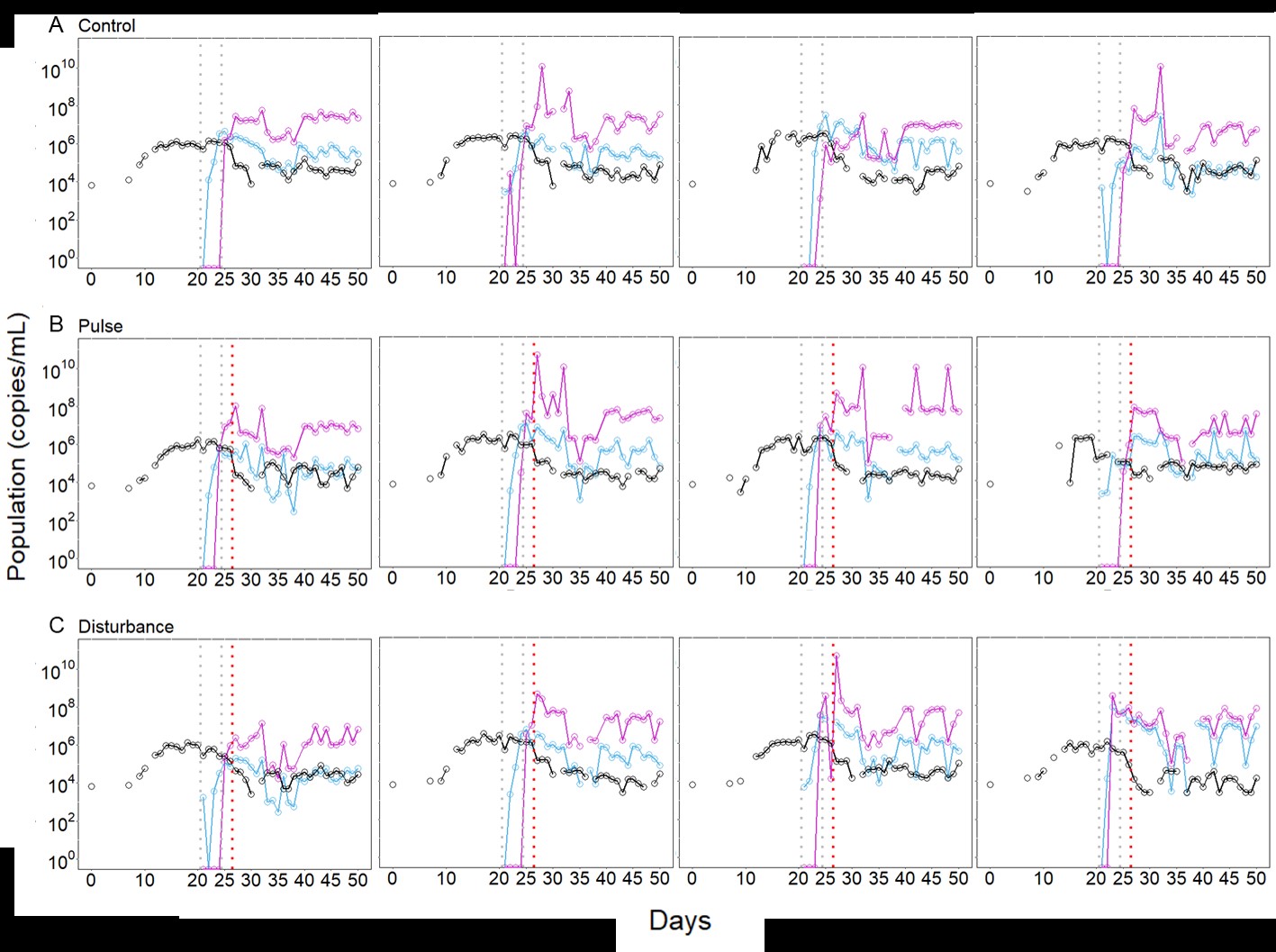

Supplement: SI_Fig_5_wrae237 [file si_fig_5_wrae237.jpeg]
